# Identification of transcriptional subtypes in lung adenocarcinoma and squamous cell carcinoma through integrative analysis of microarray and RNA sequencing data

**DOI:** 10.1038/s41598-021-88209-4

**Published:** 2021-04-22

**Authors:** François Fauteux, Anuradha Surendra, Scott McComb, Youlian Pan, Jennifer J. Hill

**Affiliations:** 1 Digital Technologies Research Centre, National Research Council Canada, Ottawa, ON Canada; 2 Human Health Therapeutics Research Centre, National Research Council Canada, Ottawa, ON Canada

**Keywords:** Cancer, Computational biology and bioinformatics, Biomarkers, Oncology

## Abstract

Classification of tumors into subtypes can inform personalized approaches to treatment including the choice of targeted therapies. The two most common lung cancer histological subtypes, lung adenocarcinoma and lung squamous cell carcinoma, have been previously divided into transcriptional subtypes using microarray data, and corresponding signatures were subsequently used to classify RNA-seq data. Cross-platform unsupervised classification facilitates the identification of robust transcriptional subtypes by combining vast amounts of publicly available microarray and RNA-seq data. However, cross-platform classification is challenging because of intrinsic differences in data generated using the two gene expression profiling technologies. In this report, we show that robust gene expression subtypes can be identified in integrated data representing over 3500 normal and tumor lung samples profiled using two widely used platforms, Affymetrix HG-U133 Plus 2.0 Array and Illumina HiSeq RNA sequencing. We tested and analyzed consensus clustering for 384 combinations of data processing methods. The agreement between subtypes identified in single-platform and cross-platform normalized data was then evaluated using a variety of statistics. Results show that unsupervised learning can be achieved with combined microarray and RNA-seq data using selected preprocessing, cross-platform normalization, and unsupervised feature selection methods. Our analysis confirmed three lung adenocarcinoma transcriptional subtypes, but only two consistent subtypes in squamous cell carcinoma, as opposed to four subtypes previously identified. Further analysis showed that tumor subtypes were associated with distinct patterns of genomic alterations in genes coding for therapeutic targets. Importantly, by integrating quantitative proteomics data, we were able to identify tumor subtype biomarkers that effectively classify samples on the basis of both gene and protein expression. This study provides the basis for further integrative data analysis across gene and protein expression profiling platforms.

## Introduction

Lung cancer is the leading cause of cancer mortality (1.8 million deaths per year globally) and although multiple treatment options are available, the five-year survival rate remains low and there is an unmet need for better therapies^1–3^. Lung cancer is a heterogeneous disease and the classification of tumors using histological and molecular features can inform personalized approaches to treatment, in particular the choice of targeted therapies^4^. The increasing use of biomarkers and targeted therapies against receptor tyrosine kinases, angiogenic factors and inhibitory immune checkpoint proteins has indeed resulted in improved patient outcomes^5–8^. Lung cancer is generally divided into small cell lung cancer and non-small cell lung cancer (NSCLC) which comprises lung adenocarcinoma (LUAD), squamous cell carcinoma (LUSC) and large cell lung cancer^9^.

The two most common NSCLC histological subtypes (LUAD and LUSC) have been classified into molecular subtypes associated with clinically relevant characteristics including prognosis and survival, oncogenic drivers, and response to targeted therapies. Transcriptional subtypes (three in LUAD and four in LUSC) were initially identified by clustering gene expression microarray data from three LUAD (total 231 patients) and five LUSC (total 382 patients) discovery cohorts^10,11^. Gene expression signatures were further applied to RNA-seq data by The Cancer Genome Atlas (TCGA) and successfully classified tumors into corresponding subtypes^12,13^. The natural extension of these analyses, namely subtype discovery in combined data from the two platforms, presents challenges because of intrinsic differences in data generated using different gene expression profiling technologies, although previous studies showed that dedicated normalization methods enabled cross-platform pattern discovery and classification^14,15^, and comparative differential expression analyses also showed good agreement between the two gene expression profiling platforms^16^.

In this study, we explored cross-platform subtype discovery in LUAD and LUSC using public gene expression data from over 3500 normal and tumor lung samples. We tested 384 combinations of preprocessing, cross-platform normalization, and unsupervised feature selection methods. The results were evaluated based on the agreement between subtypes identified in single-platform and cross-platform normalized data using various statistics including clustering comparison measures. We show that unsupervised learning can be achieved with combined microarray and RNA-seq data. We further show that tumor subtype biomarkers can be identified in integrated gene expression and quantitative proteomics data.

## Results

### Classification of lung cancer subtypes

**Figure 1:**
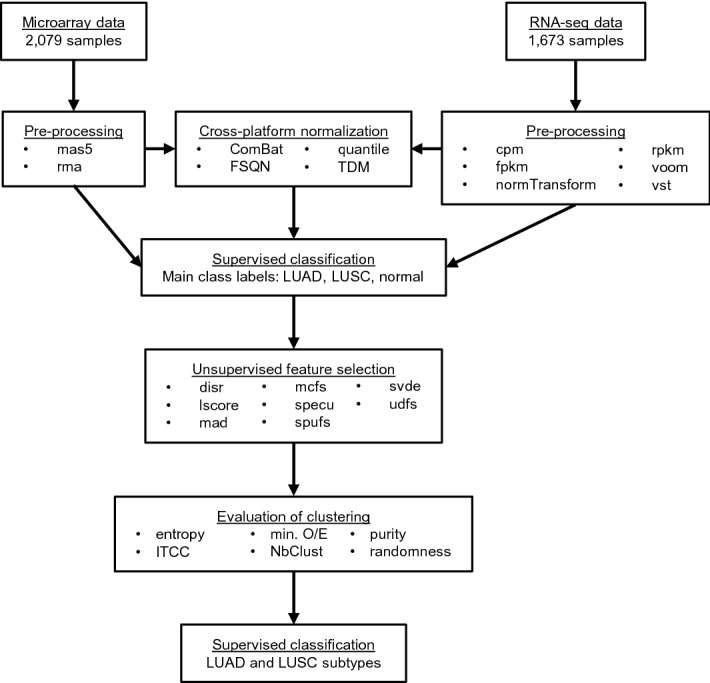
Overview of the workflow for selecting the best combination of data processing methods for cross-platform classification of lung cancer into tumor subtypes. In brief, microarray data were pre-processed using two methods, and RNA-seq data were pre-processed using six methods. Data from the two platform were combined and cross-platform normalization was performed using four methods. After filtering data by removing samples with low confidence regarding main class labels (LUAD, LUSC and normal lung), single-platform and cross-platform normalized data were submitted to unsupervised feature selection using eight methods, and then to consensus clustering. Clustering results were compared between single-platform and cross-platform normalized data using various statistics. Clustering results from the top-ranking combination of data processing methods were selected for a final round of supervised classification into tumor subtypes. Microarray pre-processing methods: mas5, microarray suite 5.0; rma, robust multi-array average. RNA-seq pre-processing methods: cpm, counts per million; fpkm, fragments per kilobase per million; normTransform, shifted logarithm transformation; rpkm, reads per kilobase per million; voom, variance modeling at the observational level; vst, variance stabilizing transformation. Cross-platform normalization methods: ComBat, empirical Bayes batch effect correction; FSQN, feature-specific quantile normalization; quantile, quantile normalization; TDM, training distribution matching. Unsupervised feature selection methods: disr, diversity-induced self-representation; lscore, Laplacian score; mad, median absolute deviation; mcfs, multi-cluster feature selection; specu, unsupervised spectral feature selection; spufs, structure preserving unsupervised feature selection; svde, singular value decomposition entropy; udfs, unsupervised discriminative features selection. Clustering statistics: entropy, platform entropy; ITCC, information theoretic clustering comparison; min(O/E), minimum observed to expected ratio; NbClust: optimal number of clusters; purity: maximum agreement between single and cross-platform data; randomness: platform randomness within clusters.

Our main objective was to identify robust expression subtypes in combined microarray and RNA-seq data for the two most common lung cancer histological subtypes (LUAD and LUSC). We collected a total of 2079 lung microarrays (500 normal, 1134 LUAD and 445 LUSC) and 1673 lung RNA-seq samples (532 normal, 640 LUAD and 501 LUSC). Although cross-platform analysis of a large number of samples can facilitate expression subtype analyses, clustering may nevertheless be sensitive to the presence of experimental (platform and batch) effects, outliers (e.g. low quality or misdiagnosed samples) as well as to data processing procedures including normalization and feature selection^17–25^. We therefore evaluated various single and cross-platform normalization and unsupervised feature selection methods to identify an optimal combination of data processing methods for cross-platform classification of lung cancer subtypes as detailed in Fig. 1.

After preprocessing single-platform data and performing cross-platform normalization, we proceeded to a first round of iterative ensemble classification (Supplementary Fig. S1) to clean data by removing a small number of samples with low confidence (< 75% of votes) regarding the main class labels (1.1–3.5% of microarrays and 0.9–1.3% of RNA-seq samples, depending on the data preprocessing method). Consensus clustering^26^ analyses were subsequently performed on expression data from a total of 384 data processing combinations, and resulting clusters were evaluated using different statistics. Cluster purity^27^ and clustering comparison measures^28^ were used to evaluate agreement between clusters identified using single-platform and combined data, while entropy^27^, a measure of association^29^ and randomness^30,31^ were used to evaluate the tendency of data to cluster by platform rather than by subtype. Lastly, we used min (O/E) , the minimum ratio of observed (size of the smallest cluster) relative to expected (number of samples divided by number of clusters), to identify data containing spurious clusters.

First, the analysis of platform entropy revealed that two cross-platform normalization methods, feature-specific quantile normalization (FSQN)^15^ and ComBat^21,32^, performed well as evidenced by their high entropy. In contrast, the two other methods tested, training distribution matching (TDM)^14^ and quantile normalization^33^, performed rather poorly showing entropy below 0.1 (Supplementary Fig. S2). For TDM, in addition to using normalized values as input, we also used raw RNA-seq counts as recommended by the authors of the TDM software^34^, which yielded similar results with entropy close to zero (data not shown). Based on these results, TDM and quantile normalization were excluded from further analyses, leaving 192 data processing combinations for each class.

Next, the following selected filters were applied: purity > 0.8 to retain only results with good agreement between single-platform and combined data, and min(O/E) > 0.1 to eliminate results containing spurious clusters. These filters had little effect on the proportion of single-platform processing or unsupervised feature selection methods in the remaining combinations; however, the vast majority of clustering results remaining after filtering were associated with three LUAD subtypes and two LUSC subtypes (Supplementary Fig. S3). To confirm this observation, we also analyzed the frequency of the best number of clusters for each dataset evaluated using the R package NbClust^35^ combined with the above filters. This analysis supported the same result showing three LUAD subtypes and two LUSC subtypes (Supplementary Fig. S4).

Remaining data processing combinations retained in both LUAD and LUSC (78 combinations) were then ranked using various statistics^27–31^. The analysis of absolute correlation between these measures revealed four groups (Supplementary Fig. S5), each of which was weighted equally for the final ranking: (1) single *vs.* cross-platform clustering agreement: purity, adjusted mutual information, adjusted Rand index, normalized information distance, normalized variation information; (2) measures related to platform entropy and platform-cluster association: entropy, Cramér’s V; (3) platform randomness within clusters: number of runs divided by sample size, rank version of von Neumann's ratio and (4) min(O/E) for minimizing the presence of spurious clusters. The top-ranking data processing combination was: microarray suite 5.0 (mas5)^36^ for microarray data, edgeR reads per kilobase per million (rpkm)^37^ for RNA-seq, FSQN^15^ for cross-platform normalization, and median absolute deviation (mad) for unsupervised feature selection (Supplementary Table S1).

**Figure 2:**
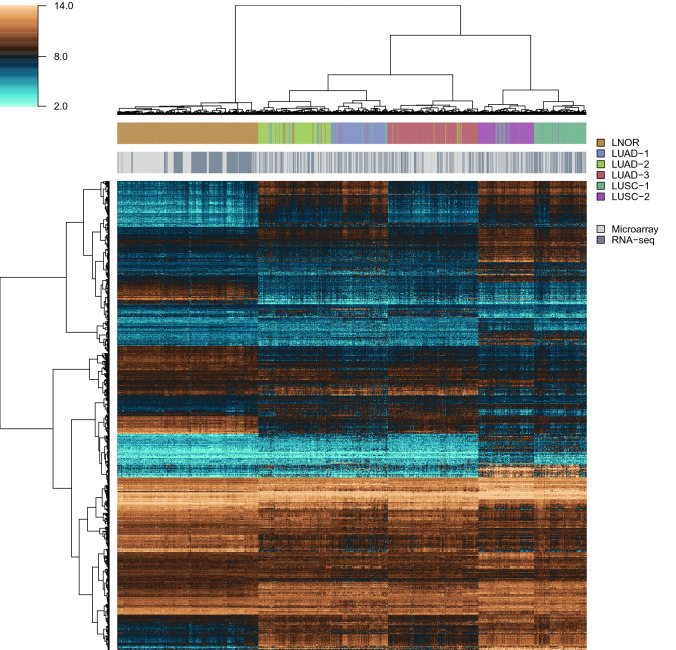
Heatmap and hierarchical clustering of lung cancer and normal lung microarray and RNA-seq data. LNOR, normal (healthy) lung; LUAD-(1–3), lung adenocarcinoma subtypes 1–3; LUSC-(1–2), lung squamous cell carcinoma subtypes 1–2. This figure was produced using R version 4.0.4 (https://www.r-project.org/).

Using clusters resulting from the top-ranking data processing combination as class labels, all lung samples (normal, LUAD and LUSC) were submitted to a final round of supervised classification, which in our experience improves the classification of some of the samples that are more difficult to classify using unsupervised methods alone. The heatmap in Fig. 2 represents the results of this final classification. Clustering of cross-platform normalized data showed consistent expression patterns with good separation between subtypes and even distribution of samples from the two expression profiling platforms for all tumor subtypes.

### Characteristics of lung cancer subtypes

To take advantage of our robust cross-platform tumor subtype classification, we proceeded to a reanalysis of LUAD and LUSC genomic alterations and patient outcomes and compared findings to those from the original studies^12,13^. Since the publication of these studies by TCGA, numerous additional patients have been enrolled and all data have been reanalyzed and harmonized to a newer version of the human genome (GRCH38)^38^ by the Genomics Data Commons (GDC)^39^.

Lung adenocarcinoma and LUSC expression subtypes from our analysis were compared to those identified in TCGA studies using annotations from TCGAbiolinks^40^. Lung adenocarcinoma subtypes 1–3 correspond to the proximal-proliferative, proximal-inflammatory and terminal respiratory unit subtypes, respectively, whereas LUSC-1 regroups the basal, primitive and secretory subtypes and LUSC-2 corresponds to the classical subtype. Analysis of available survival data (1,734 patients) revealed significant (*p* < 1e−10) differences between subtypes (Supplementary Fig. S6). Overall, LUAD-1 patients had the worst overall prognosis, whereas LUAD-3 patients had the best prognosis (both overall and relapse-free survival). For LUSC, subtype 1 had a better relapse-free survival than subtype 2. Follow-up times were relatively short for TCGA data as noted before^41^ but were in general longer for microarray data^42–47^ which strengthened the analysis for the combined survival data.

Focal copy number amplifications were assessed in each tumor subtypes using masked copy number segments from GDC analyzed with GISTIC 2.0^48^. Table 1 lists amplified focal copy number regions that overlap with genes coding for targets of clinical-stage or approved lung cancer therapeutic targets^2,5,8,49^ in any of the five lung cancer subtypes. Lung adenocarcinoma subtypes 1–2 had the highest number of focal amplifications containing potential oncogenes (ERBB2, FGFR1, KRAS, MET), whereas LUAD-3 contained none. Interestingly, LUAD subtypes 1–2 also contained KDR (*a.k.*a VEGFR) amplification, which was not reported in the original TCGA LUAD study^13^. Epithelia growth factor receptor (EGFR) was most frequently amplified in four subtypes (LUAD-1, LUAD-2, LUSC-1, LUSC-2). We further evaluated the percentage of samples carrying mutations using averages from MuTect^50^, VarScan 2^51^, Somatic Sniper^52^ and MuSE^53^ in lung cancer therapeutic targets (Table 2). Percentages of samples with mutations within each tumor subtype were highly consistent between the different variant-calling software. Again, LUAD subtypes carried the largest load of somatic mutations in potential oncogenes, and the most frequent mutated gene was KRAS. However, the LUAD-3 subtype had the most frequent mutations in EGFR. Lung squamous cell carcinoma had much less mutations as compared with LUAD, however some genes including e.g. KDR and ROS1 were frequently mutated in LUSC.

**Table 1:** Focal amplifications and therapeutic targets in LUAD and LUSC subtypes.

Class	Chromosome	Start	End	Gene
LUAD-1	chr4	54249594	58387240	KDR
LUAD-1	chr7	54467979	56385413	EGFR
LUAD-1	chr8	38413296	38619413	FGFR1
LUAD-1	chr12	25205851	25213599	KRAS
LUAD-1	chr17	39507734	39854986	ERBB2
LUAD-2	chr4	54481387	55668902	KDR
LUAD-2	chr7	116699055	116705489	MET
LUAD-2	chr7	54714092	55576700	EGFR
LUAD-2	chr12	25181421	25209325	KRAS
LUAD-2	chr17	39725021	39761258	ERBB2
LUSC-1	chr7	54751453	55698753	EGFR
LUSC-2	chr7	54699947	55357446	EGFR

**Table 2:** Percentage of samples carrying somatic mutations in therapeutic targets in LUAD and LUSC subtypes.

Gene	LUAD-1	LUAD-2	LUAD-3	LUSC-1	LUSC-2
KRAS	27.25	24.15	24.55	0.96	0
EGFR	3.89	9.12	12.93	2.03	1.13
NTRK3	11.48	9.47	2.64	4.06	3.1
KDR	10.04	7.33	4.59	6.3	4.66
PDGFRA	7.38	8.76	4.32	4.16	2.83
BRAF	7.38	6.8	6.41	1.5	2.68
ROS1	7.38	3.58	0.7	5.66	7.06
ALK	6.56	5.18	3.34	3.21	1.97
NTRK2	5.94	2.68	2.09	2.46	1.69
RET	4.92	4.65	0.69	2.67	3.81
PDGFRB	4.3	2.68	1.25	2.14	1.41
MET	2.46	4.11	2.36	0.54	0.28
NTRK1	3.28	1.79	1.25	1.6	2.96
ERBB2	0	3.22	0.56	1.39	1.13
FGFR1	0.82	0.53	0.28	0.74	0

**Figure 3:**
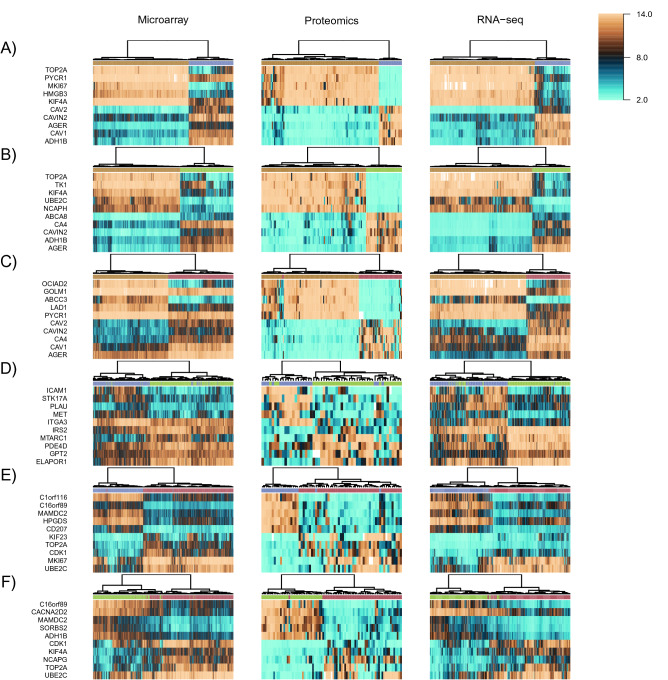
Biomarkers selected across three platforms for one-against-one classification of LUAD subtypes and normal lung. (**A**) Normal lung vs. LUAD-1; (**B**) normal lung vs. LUAD-2; (**C**) normal lung vs. LUAD-3; (**D**) LUAD-1 vs. LUAD-2; (**E**) LUAD-1 vs. LUAD-3; (**F**) LUAD-2 vs. LUAD-3. HGNC gene symbols are used to identify all biomarkers. This figure was produced using R version 4.0.4 (https://www.r-project.org/).

Because of the importance of protein assays in the clinic^54^, we further sought to integrate quantitative proteomics data with gene expression data, taking advantage of the recently released data for LUAD by the Clinical Proteomic Tumor Analysis Consortium (CPTAC)^55^. Our goal was to select a small set of biomarkers for subtype classification, capable of classifying LUAD tumors using either gene expression or quantitative proteomics data. To illustrate the robustness of the biomarkers, we combined gene expression data with proteomics data without any special cross-platform normalization other than scaling the mean and variance using microarray data as reference. Labels for CPTAC samples (205 classified samples with both RNA-seq and proteomics data) were assigned using classification of RNA-seq samples as described above. The top-10 features were selected for each one-against-one (OAO) class comparison using the log2 fold change (log2FC) and the overlap of locally adaptive kernel densities^56^. Figure 3 shows that biomarkers selected across platforms are able to accurately separate samples by clustering for all OAO class comparisons.

## Discussion

Lung adenocarcinoma and LUSC have previously been classified into transcriptional subtypes associated with important characteristics such as response to targeted therapies^10,11^. Transcriptional subtypes can further be integrated with other data such as somatic mutations and DNA methylation into multi-omics subtypes^12,13^. Previous studies have also shown that classification of NSCLC into histological subtypes can be achieved using relatively simple methods, using both microarray and RNA-seq data, for example a nearest class centroid approach using differentially expressed genes and Pearson correlation as a similarity measure^57^, or a two gene (KRT5 and AGR2) expression ratio which classified LUAD and LUSC samples with relatively high accuracy^58^. However, to our knowledge, systematic evaluation of data processing methods for unsupervised classification of LUAD and LUSC transcriptional subtypes across gene expression profiling platforms has not been performed previously. In this study, we used an unsupervised approach combining 384 data processing methods to analyze public gene expression data (2,079 microarrays and 1673 RNA-seq samples). This analysis provided insights into the optimal combination of data processing methods for cross-platform clustering, and enabled the identification of robust LUAD and LUSC expression subtypes in combined microarray and RNA-seq data.

Combinations of data processing methods were evaluated using single and cross-platform consensus clustering, and various statistics including cross-platform purity, clustering comparison measures, platform entropy, as well as randomness and min(O/E). Whereas cluster purity is generally used to evaluate the ability of a clustering method to recover known classes^27^, here it was used to evaluate the maximum agreement between single and cross-platform data. Measures of randomness enabled the evaluation of the tendency of samples to regroup by platform within clusters. In addition, the use of min(O/E) allowed effective filtering of small, spurious clusters. For unsupervised feature selection methods, the simplest and fastest method, namely median absolute deviation (mad), performed well and ranked above more complex and computationally intensive methods. The approach of binning genes by expression level also helped to avoid enrichment of features sets with low-expressed genes.

For cross-platform normalization, FSQN^15^ performed better than other methods tested in this study. This method normalizes RNA-seq data in a feature-specific manner, using microarray data as a reference (quantiles for each gene). This method performs best with larger numbers of samples, as was the case in our study. As discussed in Franks et al.^15^, its superior performance can be attributed to the fact that FSQN preserves “distribution information about the center and spread of each individual gene”. Interestingly, ComBat^21^, a method originally developed to adjust microarray data for batch effects, also performed relatively well for removing platform effects. The latter method estimates model parameters by pooling information across genes and experimental conditions. The resulting empirical Bayes estimates are used to adjust the data for unwanted sources of experimental variation. The other two methods tested for cross-platform normalization, namely quantile normalization^33^ and TDM^14^, did not perform well as evidenced by low entropy, meaning that unsupervised learning with data normalized using these methods would identify primarily platform-specific clusters. Quantile normalization ranks features using expression levels, and assigns to each feature the average value of other features with the same rank in other samples. This method can be used with a single matrix, or with a target and a reference matrix. Training distribution matching uses a similar approach, whereas target distributions are adjusted to match certain properties of a reference distribution (interquartile range, spread of the tails, extreme values) and expression data in a target sample is mapped into a range from the minimum to the maximum of the reference data. Altogether, this analysis showed that feature-specific methods such as FSQN perform better for cross-platform normalization, especially for unsupervised learning which is more sensitive than supervised approaches to experimental biases including platform effects.

After the filtering and ranking of data processing methods, we found that RNA-seq data normalized using effective gene length (edgeR rpkm^37^ and DeSeq2 fpkm^59^) performed well as input for classification after cross-platform normalization. Although RNA-seq counts normalized using gene or transcript length are generally used to compare gene expression within samples, here we show that such units are very compatible with both supervised and unsupervised classification approaches, and integrate better with microarray data for cross-platform normalization. This can be explained by the fact that methods used to summarize microarray data (mas5 or RMA) are averages across probes, and there is no direct link between gene length and expression level. RNA-seq data normalized using effective gene length are thus more similar to, and integrate better with microarray data for cross-platform normalization and machine learning tasks such as feature selection and classification.

The three LUAD subtypes identified in our analysis were highly concordant with those identified in a previous study^10^. However, single and cross-platform clustering data provided strong evidence for only two LUSC expression subtypes, in line with results from a previous study^60^, but opposed to another study that identified four subtypes^11^. This may be explained by the fact that the study by Wilkerson et al.^11^ used microarray data only, and a relatively small number of samples, although subtypes were validated across several datasets. The most limiting factor for the number of clusters was cross-platform purity, whereas only two subtypes were consistent between single-platform and cross-platform unsupervised learning in LUSC. The three subtypes previously identified within LUSC-1 (basal, primitive and secretory) may have some utility in terms of prognostics/diagnostics, but our results show that they are grouped into a single class by our data-driven, unsupervised cross-platform tumor subtype identification methodology. This is a strength in our approach, that only the most robust clusters, identified across platforms, are retained as candidate tumor subtypes. Robust classification schemes are more likely to successfully transfer to real-world applications by minimizing reliance on features that are variable due to batch or platform effects.

Each tumor subtype was analyzed separately for focal amplifications and somatic mutations in genes coding for targets of clinical-stage or approved lung cancer therapeutics, and patterns presented here show that patients within each subtype may benefit from a particular subset of targeted therapeutics. In addition, to demonstrate the robustness of transcriptional subtypes identified in this study, we showed that biomarkers accurately separating the different tumor subtypes and normal tissues can be selected and validated with mass spectrometry-based quantitative proteomics data, even though the number of proteins quantified is limited as compared with gene expression data, and generally biased towards highly expressed genes. With further validation, the identified proteins may be used as biomarkers to classify tumors using classical protein-based methods such as immunohistochemistry.

Cross-platform classification of microarray and RNA-seq data is challenging because of intrinsic differences and biases in data distribution between the two platforms. A careful selection of data processing and machine learning methods enabled cross-platform classification of lung tumor expression subtypes. Our study confirmed three LUAD expression subtypes, but only two subtypes in LUSC as opposed to four subtypes previously identified. Such classification provides insights into clinical management and drug development for LUAD and LUSC, in particular with respect to identifying subtype-specific targets for antibody-based therapeutics. This study provides the basis for further integrative analysis of microarray, RNA-seq and quantitative proteomics data, and for the classification of tumors into expression subtypes.

## Methods

### Data acquisition and preprocessing

Raw data corresponding to a total of 2,079 samples profiled using microarrays (500 normal, 1134 LUAD and 445 LUSC) were obtained from GEO^61^ and 1673 lung samples profiled using RNA-seq (532 normal, 640 LUAD and 501 LUSC) were obtained from the Sequence Read Archive (SRA)^62^ (dbGap accession number phs000424.v8.p2, fresh frozen and PAXgene-preserved samples only) and GDC^39^ (projects TCGA and CPTAC-3). All data were processed using GDC reference files (GRCh38.d1.vd1, GENCODE 22): a custom chip definition (21,552 genes) file was created for microarray data using the methods of Dai et al.^63^, and GTEx samples were re-aligned to GRCh38.d1.vd1 using GDC mRNA analysis pipeline (STAR two-pass)^64^. Microarray data were combined into one expression set and processed using R library affy^65^. RNA-seq data were combined into a single count matrix and processed using edgeR^37^, DESeq2^66^ and limma (voom)^67^ (Table 3). Reduced ranges of coding exons were used for fragments per kilobase per million (fpkm) calculations. Batch (series) effect were corrected using ComBat^21,32^. A subset of 17,095 protein-coding genes represented on both platforms was selected for further analyses. For cross-platform analysis, we first scaled the RNA-seq data to have a similar distribution (mean and variance) to that of microarray data and then merged and normalized the data from the two platforms using R libraries FSQN^15^, TDM^14^, sva^32^ and preprocessCore^68^ (Table 4).

**Table 3:** Functions used for preprocessing/normalization of microarray and RNA-seq data.

Library	Function	Description	Reference
affy	mas5	Microarray suite 5.0	^36^
affy	rma	Robust multi-array average	^93^
edgeR	cpm	Counts per million	^37^
edgeR	rpkm	Reads per kilobase per million	^59^
DESeq2	normTransform	Shifted logarithm transformation	^92^
DESeq2	vst	Variance stabilizing transformation	^94^
DESeq2	fpkm	Fragments per kilobase per million	^59^
limma	voom	Variance modeling at the observational level	^95^

**Table 4:** Functions used for cross-platform normalization of microarray and RNA-seq data.

Library	Function	Description	Reference
FSQN	quantileNormalizeByFeature	Feature-specific quantile normalization	^15^
TDM	tdm_transform	Training distribution matching	^14^
sva	ComBat	Empirical Bayes batch effect correction	^21^
preprocessCore	normalize.quantiles	Quantile normalization	^33^

### Unsupervised feature selection and clustering

Normalized data (single-platform and cross-platform) were submitted to unsupervised feature selection with eight different methods using R libraries stats^69^, Rdimtools^70^ and an in-house package for singular value decomposition entropy implemented using Rcpp^71^ (Table 5). For each dataset, a total of 2^10^ features were equally selected from eight bins delineated using gene expression means, to avoid enrichment of low-expression features which are more noisy. This number of features was deemed optimal as evaluated by cross-platform purity for a range of features between 2^8^ and 2^12^ (Supplementary Fig. S7). Data were then submitted to consensus clustering^26^ as well as evaluation of the optimal number of clusters (min = 2, max = 6) using NbClust^35^. Agreement between clusters identified using single-platform and combined data were evaluated using R package aricode^72^ as well as a custom R function to evaluate purity (maximum agreement between single and cross-platform data). The tendency of cross-platform data to cluster by platform rather than by cancer subtype was evaluated using NMF package^73^ (function entropy) and DescTools package^74^ (functions CramerV^29^, RunsTest^75^ and BartelsRankTest^31^). Spurious clusters were evaluated using min(O/E), the size of the smallest cluster over sample size divided by number of clusters.

**Table 5:** Unsupervised feature selection methods used for clustering analysis.

Library	Function	Description	Reference
Stats	mad	Median absolute deviation	^96^
	svde	Singular value decomposition entropy	^97^
Rdimtools	do.disr	Diversity-induced self-representation	^98^
Rdimtools	do.lscore	Laplacian score	^99^
Rdimtools	do.mcfs	Multi-cluster feature selection	^100^
Rdimtools	do.specu	Unsupervised spectral feature selection	^101^
Rdimtools	do.spufs	Structure preserving unsupervised feature selection	^102^
Rdimtools	do.udfs	Unsupervised discriminative feature selection	^103^

### Supervised classification

Samples were submitted to five rounds of a Monte Carlo iterative ensemble classification algorithm, modified from^56^ to include rounds of repeated random sampling (Supplementary Fig. S1). At each round, a total of 100 iterations were performed, in which 100 samples per class were randomly sampled with replacement, and classifiers were constructed for each $\left(\genfrac{}{}{0pt}{}{w}{2}\right)$ pair of classes OAO, for three supervised feature selection methods, three classification methods and six increasing number of features. All remaining samples were classified, by generating votes only where labels generated by OAO classifiers were maximal (i.e. number of classes minus one). Then, labels assigned with high confidence (> 90% of votes) by the ensemble of experts (5400 votes from 100 iterations, three feature selection methods, three classification methods, and six increasing number of features) were fed back into the data and used for subsequent feature selection and training of the classifiers. This procedure was repeated until the number of predictions was stabilized over a number of iterations (convergence was considered achieved when the number of classified samples reached a plateau). At this point, all samples with moderate to high confidence (> 75% of votes) were assigned class labels and retained for further analysis. For supervised classification purposes, filter-based feature selection was performed by selecting the top ${\left({2}^{k}\right)}_{k=5}^{10}$ features ranked using three different statistics: *q*-values derived from linear models for microarray (Limma) moderated *t*-test^76,77^, the overlapping coefficient of locally adaptive kernel density estimates^78,79^, and the weights of support vectors (WSV)^80^. Locally adaptive kernel densities and overlapping coefficients were computed using an in-house R package implemented using Rcpp^71^. The WSV were computed using the e1071 R package^81^. Classification was achieved using three algorithms implemented in the RWeka package^82^: *k*-nearest neighbors^83^, random forests^84^ and support vectors machines^85^.

### Characterization of tumor subtypes and biomarker selection

Survival data were obtained from the TCGA pan-cancer clinical resource^41^ for RNA-seq data, and from GEOmetadb^86^ for microarray data. Survival analysis was performed using the R package survival^87^ using default parameters. Masked copy number segments were obtained from GDC and processed using GISTIC 2.0^48^. The search for focal amplifications was restricted to peaks covering less than 3 Mb as recommended in Krijgsman et al.^88^. Somatic calls from MuTect^50^, VarScan 2^51^, Somatic Sniper^52^ and MuSE^53^ were obtained from the GDC, and lung tumor RNA-seq data were analyzed using VarDict^89^. These data were analyzed using the R packages maftools^90^ and VariantAnnotation^91^.

To select biomarkers for LUAD, we used microarray data processed using mas5^36^ and RNA-seq data normalized using DESEq2^92^ and log2 protein expression data from CPTAC^55^. Proteomics and RNA-seq data were scaled (mean and variance) using microarray data as reference. Features were selected to maximize the log2FC and to minimize the overlap of locally adaptive kernel densities^56^ with a total of 10 features for each OAO class comparison.

## Supplementary Information

Supplementary Figures.

Supplementary Legend.

Supplementary Table S1.

## Data Availability

Raw data analysed in this study are available to download from GEO^61^, SRA^62^ and GDC^39^ repositories. Some restrictions may apply to the availability of these data, which were used under license for the current study. Processed data analyzed as part of the current study are available from the corresponding authors on request.
