# Supplementary material for: Identification of transcriptional subtypes in lung adenocarcinoma and squamous cell carcinoma through integrative analysis of microarray and RNA sequencing data
Source: Sci Rep. 2021 Apr 22;11:8709. doi: 10.1038/s41598-021-88209-4 (PMC8062554; doi:10.1038/s41598-021-88209-4)
Supplement: Supplementary file 1 — Supplementary Figures. [file 41598_2021_88209_MOESM1_ESM.pdf]

Supplementary Information for:

Identification of transcriptional subtypes in lung adenocarcinoma and squamous cell carcinoma through integrative analysis of microarray and RNA sequencing data

François Fauteux, Anuradha Surendra, Scott McComb, Youlian Pan and Jennifer J. Hill

Supplementary Figure 1. Monte Carlo iterative ensemble classification method. LIMMA, linear models for microarray; OVL, overlap of locally adaptive kernel densities; WSV, weight of support vectors; KNN, k-nearest neighbors, RFO, random forests; SVM, support vector machine.

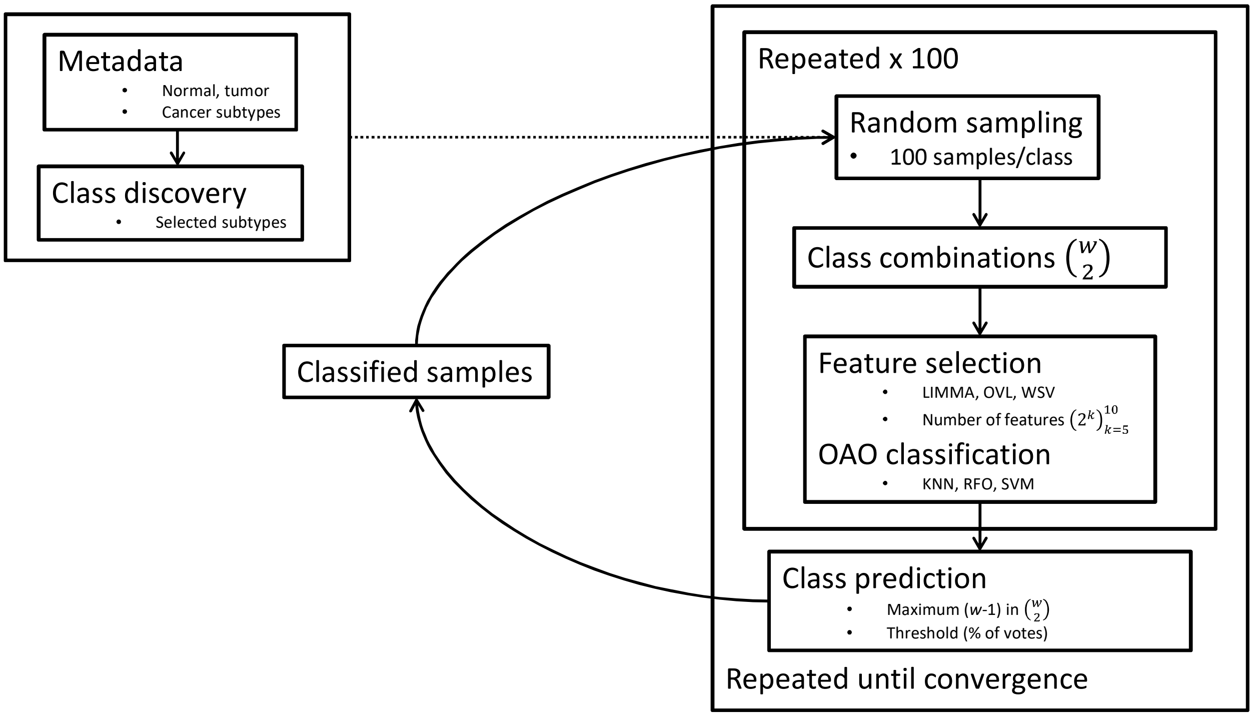

Supplementary Figure 2. Violin plot of platform entropy in LUAD and LUSC clusters for different cross−platform normalization methods. A) ComBat; B) FSQN; C) quantile; D) TDM. This figure was produced using R package vioplot version 0.3.5 (https://cran.r-project.org/web/packages/vioplot/).

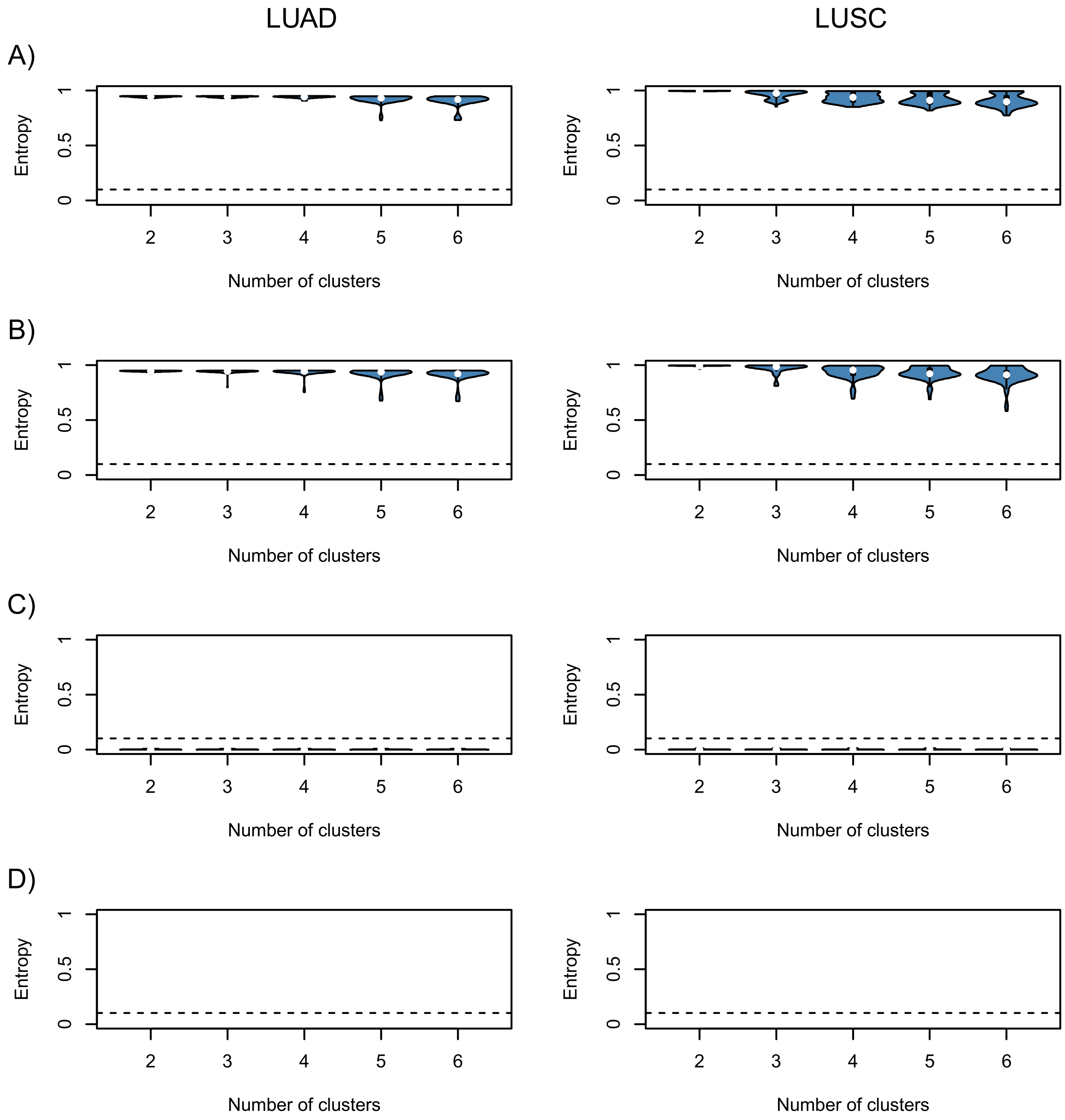

Supplementary Figure 3. Violin plot of cross−platform purity and minimum observed to expected ratio min(O/E) in LUAD and LUSC clusters. A) purity between microarray and combined data; B) purity between RNA−seq and combined data; C) min(O/E) for microarray data in cross−platform clusters; D) min(O/E) for RNA−seq data in cross−platform clusters. This figure was produced using R package vioplot version 0.3.5 (https://cran.r-project.org/web/packages/vioplot/).

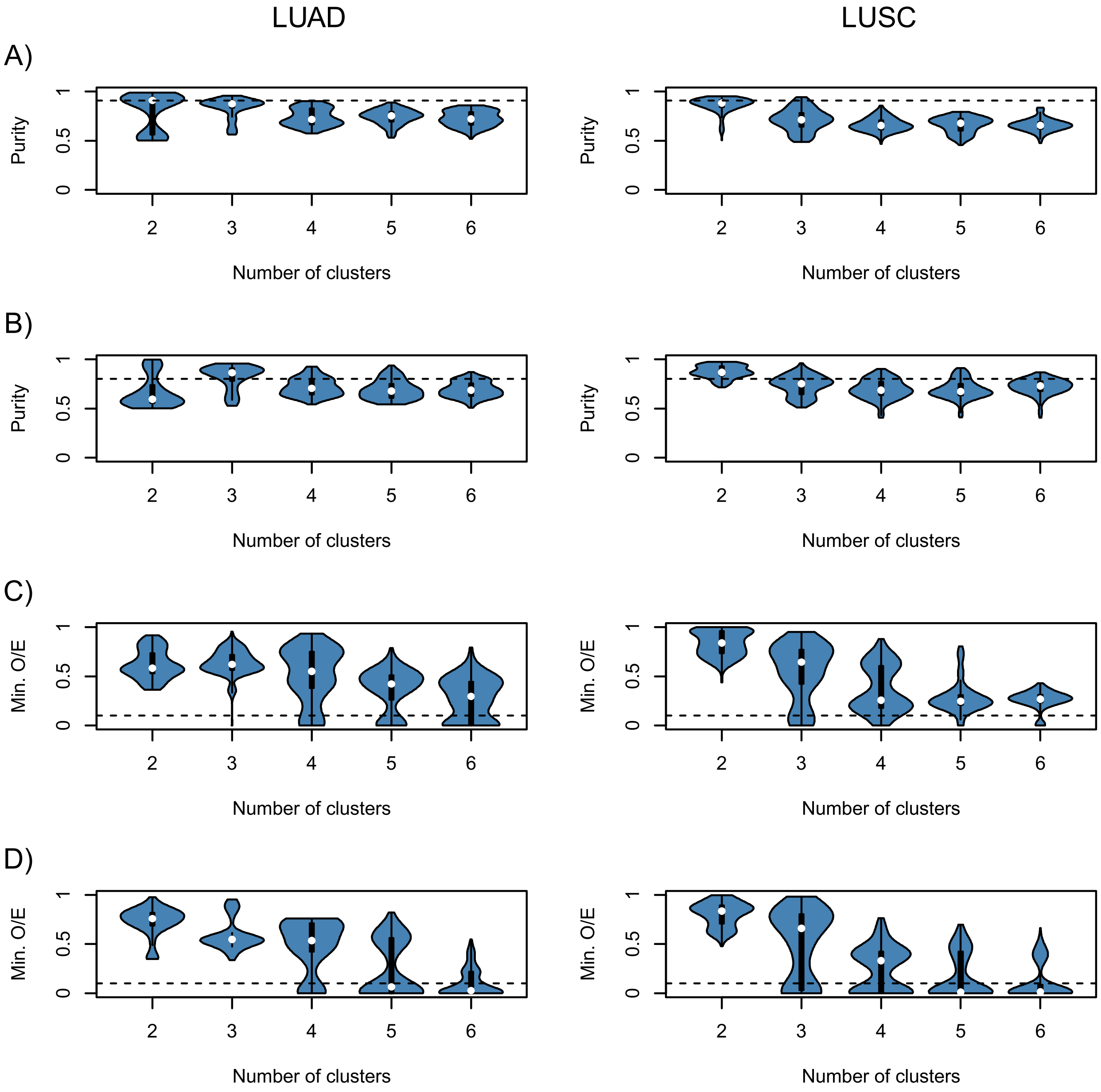

Supplementary Figure 4. Best number of clusters for LUAD and LUSC cross−platform normalized data, passing or failing filters (convergence > 0.8 and min(O/E) > 0.1).
A) LUAD; B) LUSC.

A)

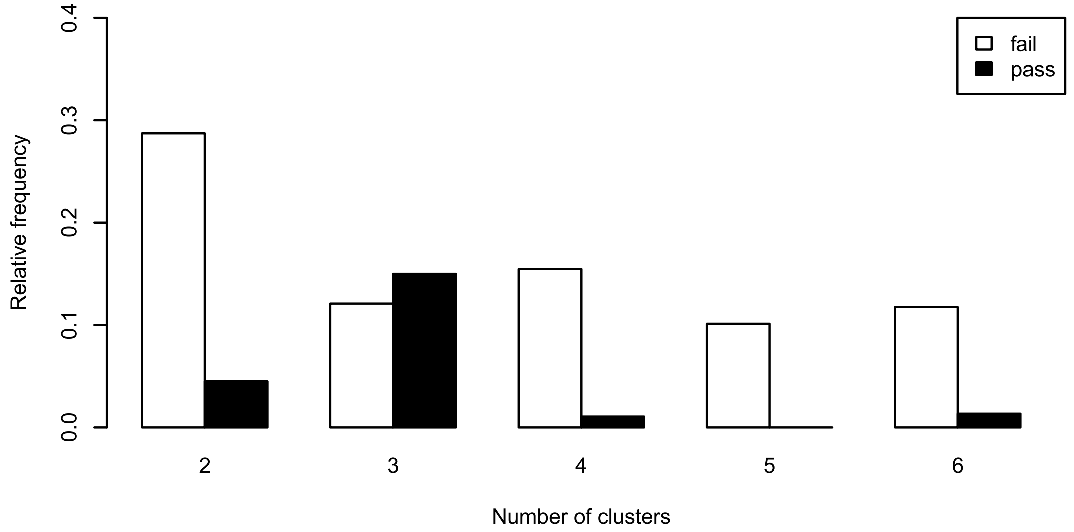

B)

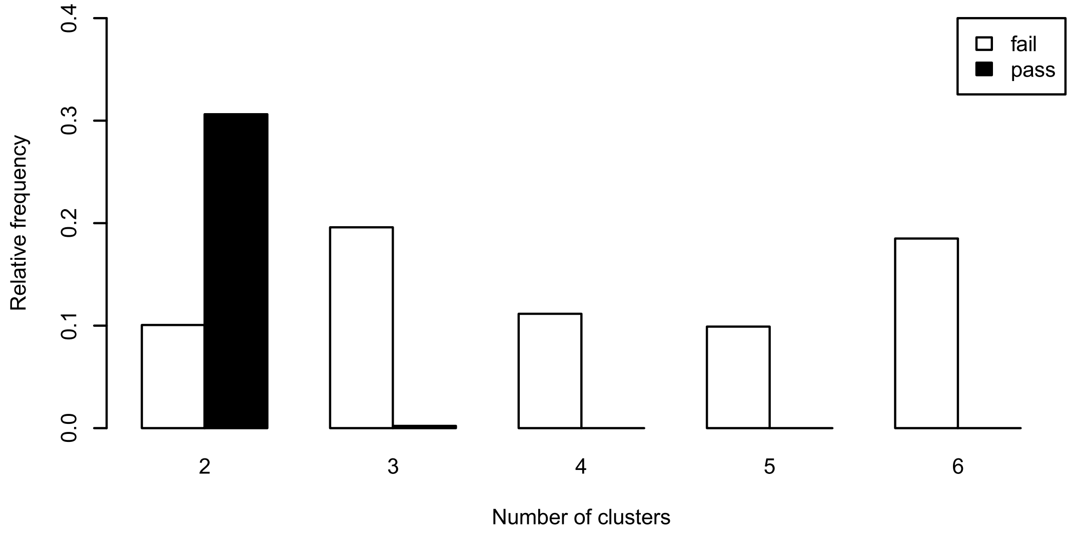

Supplementary Figure 5. Absolute correlation between clustering statistics. PTY, purity; MOE, minimum observed to expected ratio; AMI, adjusted mutual information; ARI, adjusted rand index; NID, normalized information distance; NMI, normalized mutual information; NVI, normalized variation information; ENT, entropy; CRV, Cramér's V; RPS, number of runs divided by sample size; RVN, rank version of von Neumann's ratio; MRY, microarray; RNA, RNA−seq; PLF, cross−platform. This figure was produced using R package corrplot version 0.84 (https://cran.r-project.org/web/packages/corrplot/).

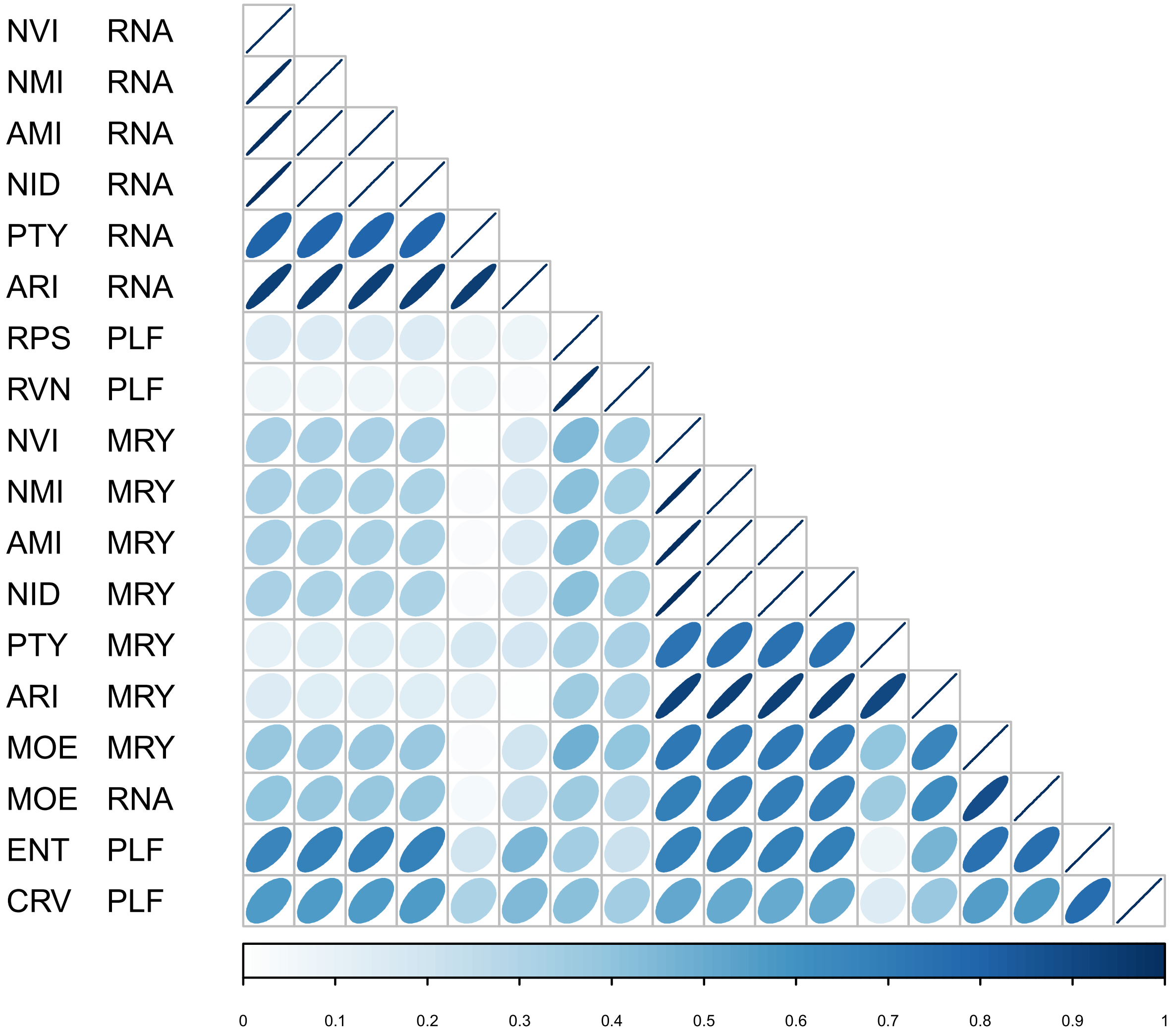

Supplementary Figure 6. Survival analysis in classified lung cancer subtypes. A) Overall, survival. B) Disease−free survival. LUAD−(1−3), lung adenocarcinoma subtypes 1−3; LUSC−(1−2), lung squamous cell carcinoma subtypes 1−2. This figure was produced using R package survival version 3.2-9 (https://cran.r-project.org/web/packages/survival/).

A)

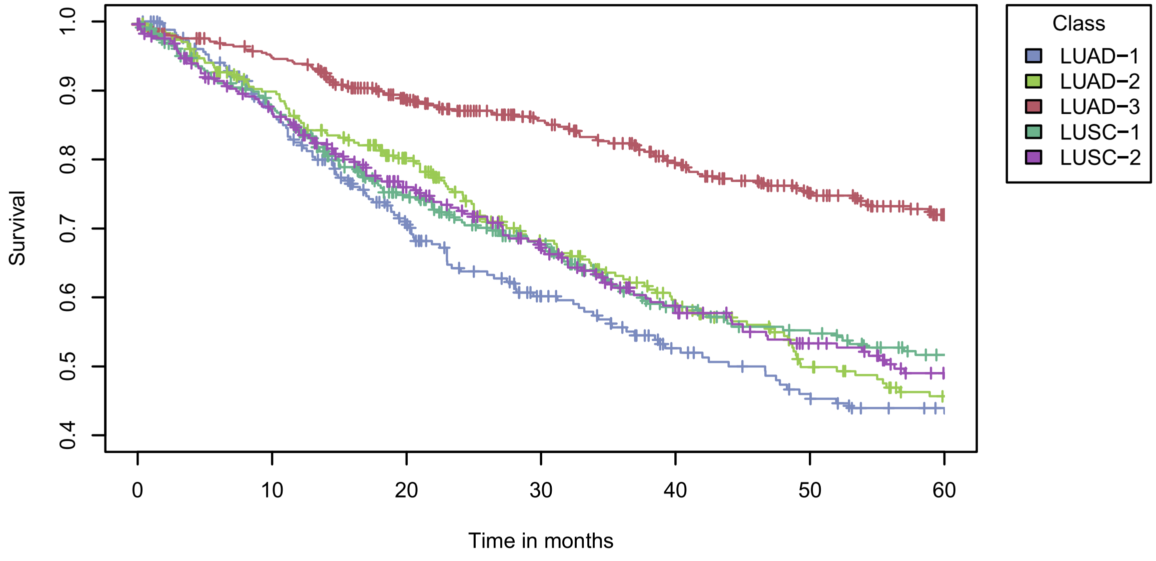

B)

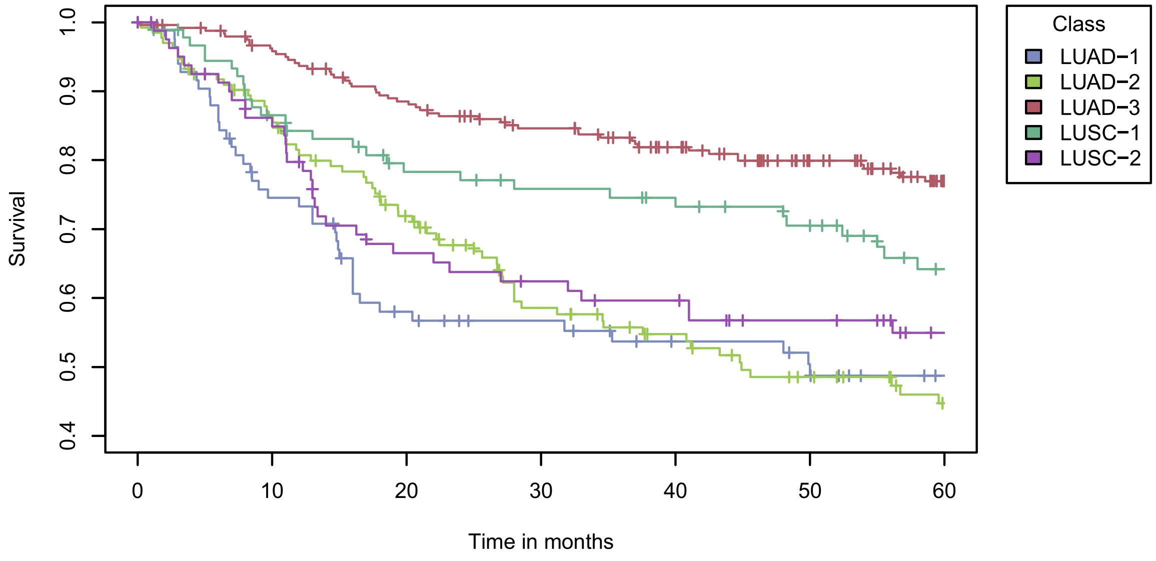

Supplementary Figure 7. Purity between single−platform and cross−platform clusters for increasing number of features (2^8 to 2^{12}).

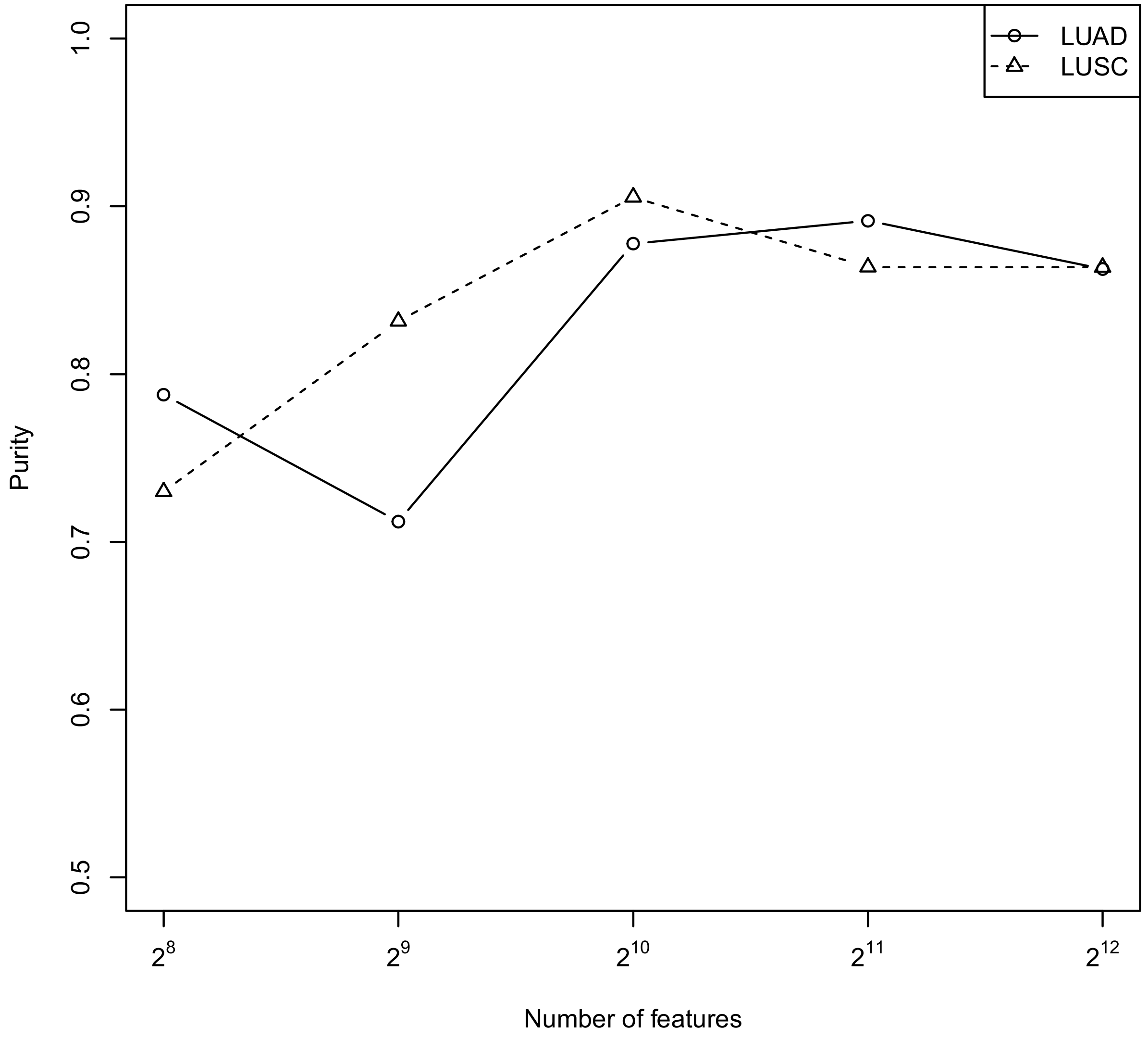

Datasets

Lung samples (normal lung, LUAD and LUSC) were selected from the following projects/series:

- Sequence Read Archive (https://www.ncbi.nlm.nih.gov/sra): SRP012682;
- Genomics Data Commons (https://gdc.cancer.gov): CPTAC-3, TCGA-LUAD, TCGA-LUSC;
- Gene Expression Omnibus (https://www.ncbi.nlm.nih.gov/geo): GSE2109, GSE3526, GSE7307, GSE8545, GSE8581, GSE10006, GSE10245, GSE10799, GSE11784, GSE12667, GSE13933, GSE15240, GSE16538, GSE18490, GSE18674, GSE18676, GSE18842, GSE18995, GSE19188, GSE19804, GSE20578, GSE20585, GSE21369, GSE21411, GSE24206, GSE27262, GSE27556, GSE27716, GSE27719, GSE28571, GSE28582, GSE30219, GSE31210, GSE31546, GSE31908, GSE33356, GSE33532, GSE33846, GSE37745, GSE37768, GSE40791, GSE43346, GSE43580, GSE50081, GSE51024.
