# Supplementary material for: Identification of transcriptional subtypes in lung adenocarcinoma and squamous cell carcinoma through integrative analysis of microarray and RNA sequencing data
Source: Sci Rep. 2021 Apr 22;11:8709. doi: 10.1038/s41598-021-88209-4 (PMC8062554; doi:10.1038/s41598-021-88209-4)
Supplement: Supplementary file 2 — Supplementary Legend. [file 41598_2021_88209_MOESM2_ESM.docx]

Supplementary Table 1. Clustering statistics. UFS, unsupervised feature selection; CPN, cross-platform normalization; PTY, purity; MOE, minimum observed to expected ratio; AMI, adjusted mutual information; ARI adjusted Rand index; NID, normalized information distance; NMI, normalized mutual information; NVI, normalized variation information; ENT, entropy; CRV, Cramér's V; RPS, number of runs divided by sample size; RVN, rank version of von Neumann's ratio.
